# Supplementary material for: Prognostic and Predictive Effects of Tumor and Plasma miR-200c-3p in Locally Advanced and Metastatic Breast Cancer
Source: Cancers (Basel). 2022 May 12;14(10):2390. doi: 10.3390/cancers14102390 (PMC9139340; doi:10.3390/cancers14102390)
Supplement: Supplementary file 1 [file cancers-14-02390-s001.zip › cancers-1718013-supplementary.pdf]

SUPPLEMENTARY MATERIALS

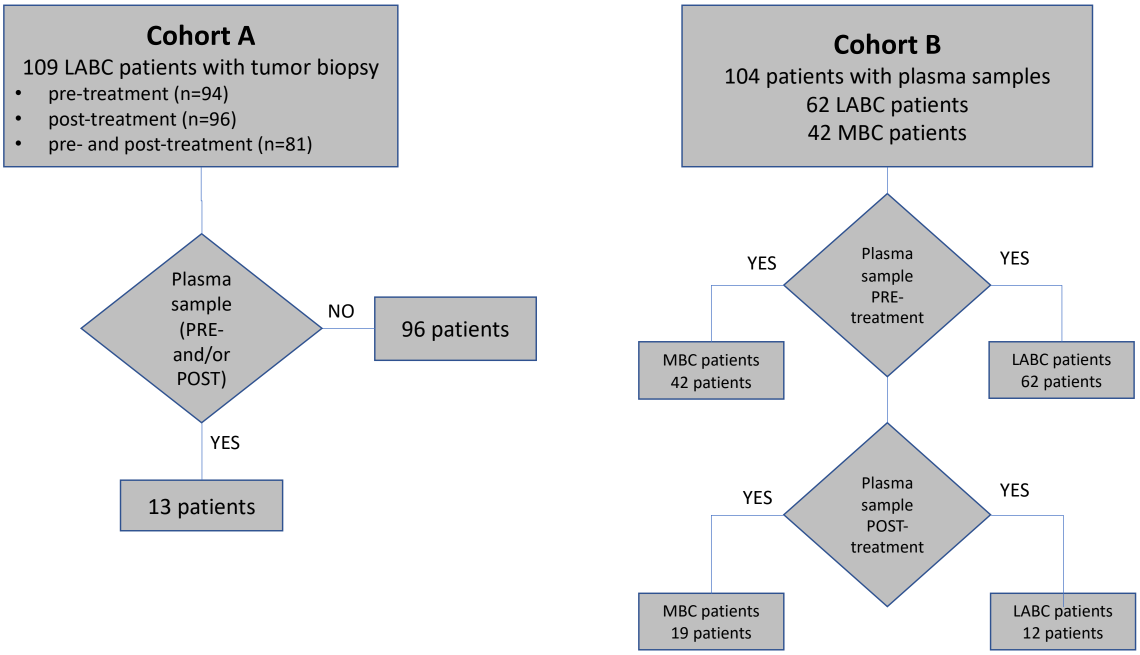

Supplemental Figure S1. Cohort diagram of the study.

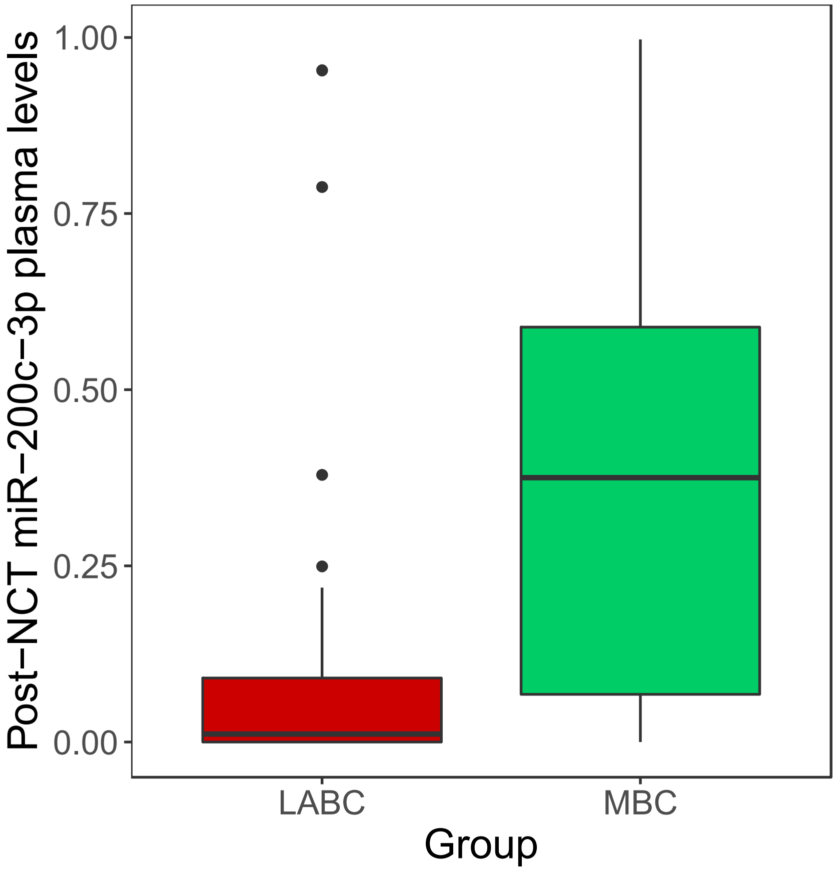

Supplementary Figure S2. Association of post-treatment miR-200c-3p plasma levels with metastatic disease.

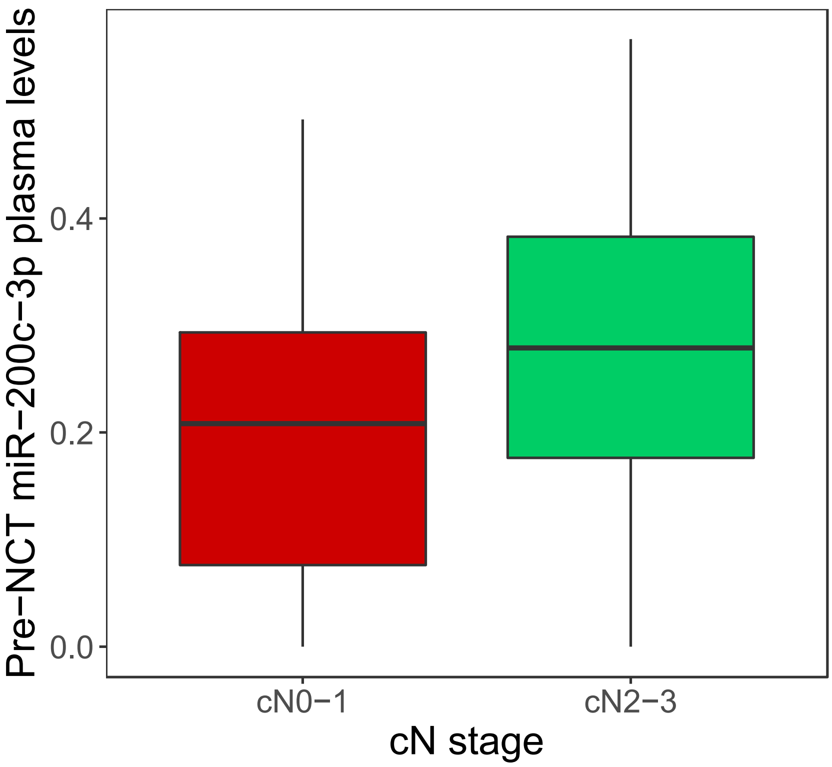

Supplementary Figure S3. Association of miR-200c plasma levels with stage cN2-3 in all LABC patients. Cohorts A and B (*n*=73).

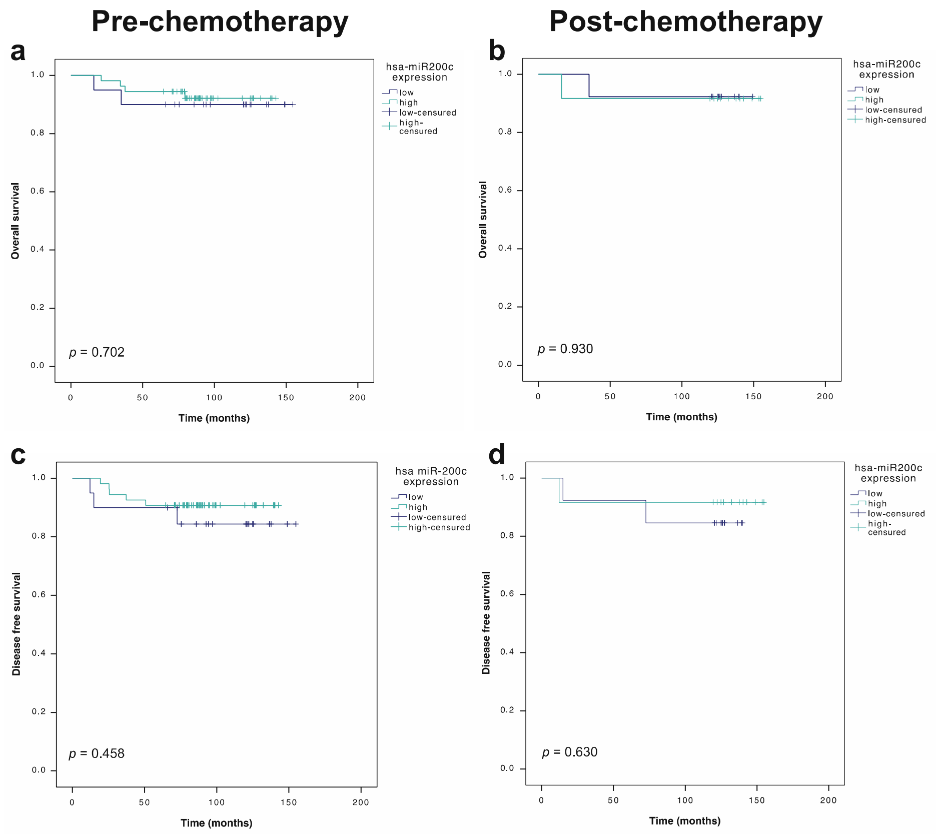

Supplementary Figure S4. Overall survival and disease free survival Kaplan-Meier curves of LABC patients according to miR-200c-3p pre-NCT and post-NCT plasma levels. Median pre-treatment and post-treatment values were used to define low and high plasma levels of miR-200c-3p. Univariate Cox regression *p* values are shown in each survival curve. (**a**) OS plot according to pre-chemotherapy miR-200c-3p plasma levels; (**b**) OS plot according to post-chemotherapy miR-200c-3p plasma levels; (**c**) DFS plot according to pre-chemotherapy miR-200c-3p plasma levels; (**d**) DFS plot according to post-chemotherapy miR-200c-3p tumor plasma levels.

Supplementary Table S1. Clinical characteristics of locally advanced breast cancer patients from cohorts A and B.

Characteristics	Category	**Cohort A** *n*=109 N (%)	**Cohort B** *n*=62 N (%)	*p*-value (Chi²)
Age	Median (range)	56 (21-79)	47 (33-80)	0.001
Histological type	Ductal	103 (94.5)	61 (98.4)	0.410
	Lobular	4 (3.7)	1 (1.6)	
	Other	2 (1.8)	0 (0)	
Histological grade	GI	6 (5.5)	2 (3.5)	0.079
	GII	35 (32.1)	30 (52.6)	
	GIII	57 (52.3)	25 (43.9)	
	N/A	11 (10.1)	5 (8.6)	
Tumor phenotype	HR+ HER2-	53 (48.6)	26 (41.9)	0.047
	HR+ Her2+	14 (12.8)	19 (30.6)	
	HR- Her2+	13 (11.9)	3 (4.8)	
	Triple negative	25 (22.9)	14 (22.6)	
	N/A	4 (3.6)	0 (0)	
cT	cT1-2	49 (44.9)	35 (56.4)	0.150
	cT3	52 (47.7)	20 (32.3)	
	cT4	7 (6.5)	7 (11.3)	
	cTx	1 (0.9)	0 (0)	
cN	cN0	37 (33.9)	17 (27.4)	0.076
	cN1	34 (31.2)	25 (40.3)	
	cN2	20 (18.3)	14 (22.6)	
	cN3	18 (16.5)	4 (6.5)	
	cNx	0 (0)	2 (3.2)	
Stage	IIA	20 (18.3)	15 (24.2)	0.237
	IIB	32 (29.4)	16 (25.8)	
	IIIA	35 (32.1)	22 (35.5)	
	IIIB	4 (3.7)	5 (8.1)	
	IIIC	18 (16.5)	4 (6.5)	
Pathologic response	pCR (ypT0/is ypN0)	22 (20.2)	12 (20.0)	0.793
	pCR breast (ypT0/ypTis)	23 (21.1)	14 (22.6)	0.821

Supplementary Table S2. Multivariate analysis of characteristics predicting pCR in LABC patients (*n*=153).

	Multivariate Analysis			
	**Beta**	**HR**	**CI (95%)**	***p*-value**
Grade	1.026	2.79	1.005-7.742	0.049
Stage III vs II	-0.473	0.623	0.252-1.539	0.305
Tumor subtype	0.547	1.729	1.190-2.511	0.004

Supplementary Table S3. Detailed first line treatment of metastastic breast cancer (MBC) patients (*n*=42).

	***n* (%)**
Endocrine-based treatment	10 (23.8)
Letrozole	9 (21.4)
Fulvestrant	1 (2.4)
Chemotherapy	18 (42.9)
Weekly paclitaxel	5 (12.0)
Capecitabine	5 (12.0)
Doxorubicin	2 (4.8)
Doxorubicin-Taxanes	3 (7.1)
(sequential)	1 (2.4)
Capecitabina-vinorelbine	1 (2.4)
Vinorelbine	1 (2.4)
Carboplatin	
Chemotherapy+biological	13 (30.9)
Taxane-Trastuzumab	5 (12.0)
Taxane-Trastuzumab-	4 (9.5)
Pertuzumab	4 (9.5)
Taxane-Bevacizumab	
No treatment *	1 (2.4)

* One elderly patient with HER2-HR- MBC was not treated due to poor performance status and relevant comorbidity.

Supplementary Table S4. Normalized miR-200c-3p plasma levels in locally advanced breast cancer (LABC), MBC and controls for each cohort.

	**Median**	**IQR**
Control	0.14	0.09-0.22
LABC (combined)	0.23	0.11-0.35
LABC (cohort B)	0.26	0.14-0.37
LABC (cohort A)	0.06	0-0.29
MBC (cohort B)	0.23	0.18-0.53

Supplementary Table S5. Association between pre- and post-NCT miR-200c-3p tumor expression in primary tumor (pre-treatment, *n*=94; post-treatment, *n*=77) and plasma (pre-treatment, *n*=13; post-treatment, *n*=8) and clinicopathological features in LABC patients (cohort A)

		**miR-200c-3p Primary Tumor (pre-treatment)**	***p*-value Primary Tumor (pre-treatment)**	**miR-200c-3p Plasma (pre-treatment)**	***p*-value Plasma (pre-treatment)**	**miR-200c-3p Primary Tumor (post-treatment)**	***p*-value Primary Tumor (post-treatment)**	**miR-200c-3p Plasma (post-treatment)**	***p*-value Plasma (post-treatment)**
Menopausal status at diagnosis, *n*	Postmenopausal	51	0.847	5	0.882	36	0.232	2	1
	Premenopausal	43		8		41		6	
Lymph node clinical stage, *n*	cN0-1	61	0.022	9	0.008	53	0.027	6	0.252
	cN2-3	33		4		24		2	
Histological Grade, *n*	G I	5	0.452	0	0.863	6	0.261	0	0.558
	GII	32		4		29		4	
	GII	49		8		33		3	
	Not valuable	4		0		4		0	
Tumor clinical stage, *n*	cT1-2	44	0.15	5	0.553	37	0.195	5	0.124
	cT3-4	50		8		40		3	
Tumor phenotype, *n*	HR+ Her2-	45	0.833	4	0.494	47	0.054	3	0.482
	HR- Her2+	12		2		10		2	
	Her2+	12		4		4		0	
	TNBC	23		3		15		3	
Pathological complete response (pCR), *n*	No	75	0.379	8	0.711	-	-	-	-
	Yes	19		5		-	-	-	-
Post-NCT nodal stage (ypN), *n*	Negative	50	0.289	9	0.938	35	<0.001	4	0.321
	Positive	44		4		42		4	
